# A Treatise on Cancer, and Its Treatment

**Published:** 1857-09

**Authors:** 


ART. III.—*A Treatise on Cancer, and Its Treatment.* By J. WELDON FELL, M.D., of the University of New York. London: James Churchill. 8vo. pp. 95.

IN a treatise, so called, on cancer, consisting of but sixty-three pages of text, of which fifty-six are made up of an indifferent compilation on the varieties of the disease and their treatment—leaving only seven pages for the author to unfold his curative plan—little can be expected in the way of pathological research, and of the rationale and details of therapeutical treatment. We say therapeutical, for it is one aim of the author to show the superiority of his remedial course to the surgical one. Thirty-two pages are taken up with selected cases. Were the present treatise the work of a regular physician, and its contents a frank and willing offering made to his professional brethren, little would be left for analysis and criticism, beyond a mention of any peculiarity of treatment pursued by the author, and a comparison of it with the experience and observations of preceding and contemporary writers. But, unhappily, neither of these conditions have been observed by Dr. Fell. He went to London, and announced himself to be the possessor of a new and successful method of treating cancer: but the articles with which he worked were kept secret; so that he alone could perform the cure; and the poor sufferers from this terrible disease, in all parts of the world, were left to linger and die, except those who employed him; and how few, under the most favorable circumstances, could reach him, or be reached by him. This much for the part of mystery which he enacted. That of publicity was forced on him by what he would call a premature discovery of his remedies, or rather remedy. His fellow-men, therefore, owe him no thanks for giving *now*, the details of a treatment which they would have learned from other quarters; and which he had previously wished to make a matter of gainful trade by selling his secret to the British Government. But Parliament, which held the purse-strings, refused the offer. Dr. Fell was then taken up by the Surgical Staff of Middlesex Hospital, and one of its wards was placed under his exclusive control, for the treatment of cases of cancer.

The countenance thus given to an American empiric in London, by the surgeons of one of its hospitals, might be construed into an acknowledgment and defence of the correctness of his course. It is simply an increase of the original wrong, by a greater number becoming concerned in it. A swindler will not, we believe, be looked on with a more indulgent eye, or receive a lighter punishment, because he may have had numerous abettors at the time, and accessories after the act, who, from the moment they laid themselves open to a criminal prosecution, lost their privilege of appearing as witnesses in his favor. Their testimony, in the eye of the law, would be as worthless as his own for palliating or excusing his crime. So, also, the moral obloquy, unavoidably associated with the practice of quackery, attaches not only to the chief quack—who originated the thing—but also to the aid-quacks, who make themselves such by becoming his apologists and defenders. These persons join him both in infringing the moral code, and in conspiring against the public health.

We are sorry to be obliged to use such plain language, but the times require it. When we find some men of intelligence who would resent an insinuation against a want of fairness in their daily transactions, as well as members of the clerical profession, and worse still, members of our own, to be so purblind as not to see this matter in its true light, it is high time to determine whether there are recognized tests of sound mental vision, and whether they, or we who speak in the name of the great body of the medical profession, are looking at a phantasmagoria, instead of things in their true measure and proportion? Is their moral sense obtuse, or ours painfully acute? If falsehood, often in the very title of the quack remedies, as well as in the announcements of their general properties and composition, and of their immediate and remote effects; if deception in withholding all public knowledge, not only of their nature and composition, but also of the circumstances under which they must necessarily prove injurious; thus deluding thousands in every community into a belief of the entire safety, as well as the benefit, with which they may be used by all persons, at all times; if, we say, these be praiseworthy acts, entitled to the countenance and support of truth-loving, honest, and religious men, there is an obvious necessity for a new standard of religion, and a new code of morality, different from those which have hitherto been thought binding.

Will it be alleged that the object is so praiseworthy that some solecisms in ethics must be pardoned in the use of the means for attaining it. The plea would be invalid even if the means led, of a certainty, to the promised results. It is such a plea as would justify the poor for snatching from the rich, the hungry from the overfed, a portion of their wealth and their abundance. It would justify a man for having recourse to all kinds of fraud in order to support a large and helpless family; or a son for robbery with violence to enable him, by the proceeds, to save a mother from utter destitution and famine. But the case is not fairly stated when it is assumed, that the primary and main object of a man who makes and vends secret remedies, or pretends to a secret method of cure, is to benefit the health of his fellow-creatures. His object is to make money. He engages in a speculation for the purpose, and he uses every art, every trick, every deception, to sell his goods, a knowledge of the nature, fabric, or composition of which he withholds from his purchasers. He asks for their unlimited confidence in his assertions of the purity of his intentions, and of his wisdom, and intimate knowledge of the animal economy, and of the operation of medicines—he, a mere adventurer, who probably never read a book on these subjects, culled a simple, or performed an experiment, and who is obliged to get a hireling as unprincipled as himself, to write for publication the stuff and jargon which is to impose on the public, and to which are afterwards appended certificates of doctors of medicine, of law, and of divinity. Sometimes, and here is the greatest grievance, a physician of some education comes out as quack-in-chief, oblivious of or disregarding all ethical obligations, and the long line of examples of disinterestedness and pure benevolence on the part of medical men. Just now Dr. Fell has won for himself this hateful notoriety. But his secret is made public, and he has himself become the historian of its composition and effects. A brief notice of both will suffice, as there is neither novelty in the means employed nor success from their use to justify a more extended one.

The active article in the compound which Dr. Fell employed as a secret remedy for eighteen months, in the treatment of cancer, is Chloride of Zinc. He combined it with Sanguinaria Canadensis, and added wheat flour and water to form the whole into a paste, as in the following formula:

℞—Sanguinaria Canadensis, ℥ss vel ℥i.
Chlor. Zinci, ℥ss. vel ℥i.
Aquæ, ℥i.
Pulv. Sem. Tritic. Hibern. q. s.
Mix and form a paste the consistence of treacle.

From the prominence and priority of place which he gives to the Sanguinaria (or blood root, so called from its color), the puccoon of the Indians, the reader might be led to infer that it was the article on which the author placed the most reliance in his cancer-curing process. The narrative, detailing his first thoughts on the conditions for the removal of cancer and his vain efforts, for a while, to produce the desired effects, until he heard of a root used by the North American Indians, which he was told by traders had been given with success in this disease, is written in the usual and almost stereotyped style of nostrum-mongers and manufacturers of "Balm of Gilead," "Panacea," &c. A dream, in which the wonderful virtues of this plant was revealed, is all that is wanting to place the narrative of Dr. Fell on a par with the highest productions of quack literature. After mentioning the great activity of the Sanguinaria and its use in various diseases, he tells of his experiments with it on ulcerative surfaces, which ended in a removal of the tumor and the cure of the patient. The connection between the tumor and the ulcerated surfaces is not explained. Months were, however, required before the desired result was obtained; and the next aim of Dr. Fell was to combine various substances with it so as to hasten its action. None of these appeared to do as well as the chloride of zinc, "for with this compound large ulcerated tumors were removed in a few weeks, with comparatively little, and in many cases, no pain; at the same time obtaining, by absorption and by the internal use, all the good effects of the puccoon.

"The next object was to adapt the treatment to non-ulcerated tumors, and, as a preliminary step, the cutis was destroyed by nitric acid, and the paste applied; but it was found that the eschar, produced by such application, was so thin that it would require a long time to remove a large tumor.

"Incisions about half an inch apart were then made through the eschar, avoiding the living tissues, and the paste spread upon strips of cotton inserted into them daily; this plan succeeded admirably, and is *believed to be entirely original.*

"It was then found that, although the action of the puccoon was much assisted by the addition of the zinc, yet it was slow enough to allow its complete absorption, thereby enabling it to exert its peculiar constitutional effects, and, at the same time, removing the diseased mass in a few weeks."

We have given the sum and substance of Dr. Fell's plan of treatment of cancer, as far as his claims to originality or novelty go. The author may possibly believe in what he writes of the curative powers of the Sanguinaria in this disease, and of its being the chief active ingredient in his paste, while the chloride of zinc is but an auxiliary—an important one he admits. For ourselves, we have no hesitation in reversing the order of the relative degrees of importance of the two articles which the author lays down, and in expressing our entire belief that the chloride of zinc is the active ingredient, and the Sanguinaria but auxiliary, and that to a very limited extent. Our opinion coincides so entirely with that of one of our London contemporaries (*Med. Times and Gazette*, June 6), as will be seen in the following extract:

"Now there are certain passages in this statement of Dr. Fell which we cannot permit to pass unchallenged. First, as to the *Sanguinaria*, we have not the slightest hesitation in expressing our conviction that the Sanguinaria has little or nothing to do with the results of the application, and that the chloride of zinc is the only active agent. The effects of the caustic, as described by the Surgeons of the Middlesex, are precisely those described by Canquoin, Maisonneuve, and others who have used the chloride of zinc in paste in France; they are precisely those observed years ago by Sir Benjamin Brodie, and more lately by Mr. Haviland and Mr. Moullin in this country; they are also very similar to those obtained by Mr. Stanley at St. Bartholomew's by the use of dilute solutions of the chloride of zinc. The Sanguinaria does not appear to be even as useful as the ranunculus and coltsfoot mixed with the arsenical paste used in the last century by Plunket and Guy. This had the effect of blistering the skin, and doing away with the necessity of cauterizing it with nitric acid after the fashion of Dr. Fell; but the Sanguinaria used in Dr. Fell's formula with the chloride of zinc, though it may possibly have some sedative or astringent action, is in all probability chiefly retained as a coloring matter, and as a drug not easily procurable in this country."

The writer farther shows, that his claim to originality in the insertion of the paste into the incisions which Dr. Fell made into the eschar, is without foundation. Mr. Justamond, in an account of the treatment of cancer, published in 1780, points out a course of proceeding identical with that recommended by Dr. Fell, as far as relates to incisions, and the introduction of the paste into them. Mr. Justamond used lunar caustic and arsenical powder, where Dr. Fell used nitric acid and chloride of zinc.

A man who has once entered on the devious road of empiricism knows not where to stop nor into how many windings he will be led; nor have others any certain standard by which to measure or anticipate the course he may pursue. In the whole narrative of the steps by which he was led to the adoption of his plan of healing cancer, as well as in his statements of the comparative effects of the ingredients, the vegetable and the mineral, there seems to us to be a design on the part of the author to mislead the professional and general public by the attribution of curative virtues to the Sanguinaria, which are neither proved nor probable. By implication he sins against truth, as, in a more open and direct manner, Dr. Martin, another of our American cancer doctors, did, some seventy years ago, by his repeatedly assuring Dr. Rush that his powder "was wholly a vegetable preparation." Said powder was composed of oxide of arsenic with the addition of some vegetable substance, as ascertained by experiments instituted for the purpose by Dr. Rush. This is a specimen, by no means an uncommon or unusual one, of the veracity of quacks when they speak of the nature and composition of their remedies. Dr. Rush, in his remarks on the use and effects of Martin's powder and of the mode of proceeding in the treatment of cancerous tumors by its compounder, says: "In these cases, where Dr. Martin used to extract cancerous or scirrhous tumors, that were not ulcerated, I have reason to believe that he always broke the skin with Spanish flies." In this feature of the treatment, we find a precedent for that by Dr. Fell, in the use of nitric acid for the same purpose. We cannot, indeed, resist a belief that he had read Dr. Rush's paper,* and turned to trading account the lessons of deception taught by Martin.

To the occasional use of other local remedies, such as the sulphuret of zinc with sanguinaria, myrtle wax, watery extract of opium, and extract of conium, and spermaceti ointment, and another ointment consisting of iodide of lead, glycerine, and spermaceti ointment, employed by Dr. Fell, it is enough for us barely to allude. On a more important branch of the subject, a change in the cancerous diathesis, by remedies acting on the constitution, he imparts no information worth noticing. His directions "for a nourishing and sustaining diet" are common-place, and the addition "of giving the puccoon in small and repeated doses," can have no other effect than keeping alive the hopes of the patients, if they are made to believe that they are using a new and powerful medicine.

We shall repeat the words of the London Lancet (June 13th), which gives credit to the author of the Treatise on Cancer, for sense at the expense of his sincerity, as follows: "In fine, let us say of Dr. Fell, that we do not believe him to be so silly as to suppose that his remedies really possess the powers which he desires the public to believe." An analysis of even his selected cures, thirty-five in number, out of seventy-five in his notebook, will not bear him out in his assertions of his having discovered a successful method of curing cancer. The period between July, 1855, from which he dates, and the time of writing his treatise, is too short to enable him to speak with any confidence of a permanent cure, or to assert that the disease will not return. His very first case ended fatally in a few months

* An account of the external use of arsenic in the cure of cancer, in Med. Inq. and Observ., Vol. I.

after the *second* cure of the cancer. The subject was a lady, who died of "a pulmonary difficulty," most probably a cancer of the lungs or pleura.

The report of the Surgical Staff of the Middlesex Hospital, dated March 18, 1857, and signed by Alexander Shaw, Campbell De Morgan, Charles H. Moore, and Mitchell Henry, is written in the discreditable and illogical fashion of the usual certificates in favor of quacks and their remedies, and entitles its authors to the contumely which attends such productions. "Considering," to use their own language, "the limited period which has elapsed since the treatment in the hospital was commenced (Jan. 22d)," a few days over two months, common modesty and discretion ought to have kept "the Surgical Staff" from making fools of themselves in this way. Why should they desire any addition to the bad notoriety which they had already acquired by colluding with an empiric, to the extent of allowing him to carry on his plan of secret treatment in one of the wards of their hospital?

